# Unraveling Admixture, Inbreeding, and Recent Selection Signatures in West African Indigenous Cattle Populations in Benin

**DOI:** 10.3389/fgene.2021.657282

**Published:** 2021-12-08

**Authors:** Sèyi Fridaïus Ulrich Vanvanhossou, Tong Yin, Carsten Scheper, Ruedi Fries, Luc Hippolyte Dossa, Sven König

**Affiliations:** ^1^ Institute of Animal Breeding and Genetics, Justus-Liebig-University Gießen, Gießen, Germany; ^2^ Chair of Animal Breeding, Technische Universität München, Freising-Weihenstephan, Germany; ^3^ School of Science and Technics of Animal Production, Faculty of Agricultural Sciences, University of Abomey-Calavi, Abomey-Calavi, Benin

**Keywords:** Dahomey cattle, genomic inbreeding, admixture, haplotype, BoLA genes, adaptation

## Abstract

The Dwarf Lagune and the Savannah Somba cattle in Benin are typical representatives of the endangered West African indigenous Shorthorn taurine. The Lagune was previously exported to African and European countries and bred as Dahomey cattle, whereas the Somba contributed to the formation of two indigenous hybrids known as Borgou and Pabli cattle. These breeds are affected by demographic, economic, and environmental pressures in local production systems. Considering current and historical genomic data, we applied a formal test of admixture, estimated admixture proportions, and computed genomic inbreeding coefficients to characterize the five breeds. Subsequently, we unraveled the most recent selection signatures using the cross-population extended haplotype homozygosity approach, based on the current and historical genotypes. Results from principal component analyses and high proportion of Lagune ancestry confirm the Lagune origin of the European Dahomey cattle. Moreover, the Dahomey cattle displayed neither indicine nor European taurine (EUT) background, but they shared on average 40% of autozygosity from common ancestors, dated approximately eight generations ago. The Lagune cattle presented inbreeding coefficients larger than 0.13; however, the Somba and the hybrids (Borgou and Pabli) were less inbred (≤0.08). We detected evidence of admixture in the Somba and Lagune cattle, but they exhibited a similar African taurine (AFT) ancestral proportion (≥96%) to historical populations, respectively. A moderate and stable AFT ancestral proportion (62%) was also inferred for less admixed hybrid cattle including the Pabli. In contrast, the current Borgou samples displayed a lower AFT ancestral proportion (47%) than historical samples (63%). Irrespective of the admixture proportions, the hybrid populations displayed more selection signatures related to economic traits (reproduction, growth, and milk) than the taurine. In contrast, the taurine, especially the Somba, presented several regions known to be associated with adaptive traits (immunity and feed efficiency). The identified subregion of bovine leukocyte antigen (BoLA) class IIb (including *DSB* and *BOLA-DYA*) in Somba cattle is interestingly uncommon in other African breeds, suggesting further investigations to understand its association with specific adaptation to endemic diseases in Benin. Overall, our study provides deeper insights into recent evolutionary processes in the Beninese indigenous cattle and their aptitude for conservation and genetic improvement.

## Introduction

Western Africa represents a reservoir of the unique and diverse African animal genetic resources, due to a complex history including migration, dispersion, natural and artificial selection, and crossbreeding (Hanotte et al., 2002; Stock and Gifford-Gonzalez, 2013). The region is the exclusive current habitat for indigenous taurine cattle (*Bos taurus*) on the continent (Mwai et al., 2015). African humpless taurine cattle are the earliest known cattle populations on the continent according to historical and archaeological evidence (Blench and MacDonald, 1999). These animals share early common ancestors with European taurine (EUT) cattle dated to before the domestication process (Ho et al., 2008; Murray et al., 2010). They are subdivided into two subgroups, the Longhorn and the Shorthorn taurine. The origins of the two populations are still controversial, but it is scientifically accepted that they have been separately introduced into West Africa several millennia BC (Epstein, 1971; Payne and Hodges, 1997; Hanotte et al., 2002). The N’Dama is the unique reported Longhorn taurine breed, whereas the humpless Shorthorns include the Savannah breeds (Baoulé, Doayo, and Somba) and the Dwarf forest breeds (Dwarf Muturu, Liberian Dwarf, and Lagune), which are barely characterized across West African countries (Rege, 1999).

The Somba and Lagune represent indigenous African Savannah and Dwarf taurine in Benin, respectively. Both are characterized by stocky animals, resistant to diseases (especially trypanosome), and able to survive and produce in harsh environments (Dossa and Vanvanhossou, 2016; Ahozonlin et al., 2020). The Somba cattle are the typical ancestral residue of West African Savannah Shorthorns that migrated to Togo, Ghana, and the Ivory-coast (Rege, 1999). They have been preserved from admixture until recent past in the Atacora mountain area in Northwest Benin (Hall et al., 1995; Rege, 1999). The Lagune cattle are present not only in Southern Benin but also in the coastal areas and near lagoons in West and Central Africa, as indicated by their name (Rege et al., 1994). The Lagune are described as the smallest of the African taurine (AFT) cattle (93 cm of average height at withers) and acquired their Dwarf phenotype through adaptation to environmental constraints in their belt (Rege et al., 1994). According to previous studies, this breed is genetically different from other Shorthorn cattle breeds (Moazami Goudarzi et al., 2001; Gautier et al., 2009). Berthier et al. (2015) reported higher trypanotolerance with lesser anemic condition in Lagune animals in comparison to the Baoule. However, genomic regions affected by divergent selection and environmental adaptation of the Lagune and the Somba remain unknown.

The Lagune cattle from Benin had been exported during the early 20th century (around 1904) to different African countries such as Zaire (current Democratic Republic of Congo), Zambia, Gabon, as well as to Europe where they are known as Dahomey cattle (Rege et al., 1994; Porter et al., 2016), because Dahomey is the former name of the country of Benin. The European Dahomey cattle are presently kept and bred by a breeder association involving 77 farmers (Verband Europäisches Dahomey-Zwergrind, 2019) from four European countries including Germany, Austria, Czech Republic, and Switzerland (http://www.dahomey-zwergrind.com). To date, neither scientific study nor census addressed these animals. According to the breeder association, the current population is characterized by a small size (adult body weight between 150 and 300 kg and sacrum height from 80 to 105 cm), short horn, easy calving, and good temperament. These characteristics are similar to those of the original Lagune population in Benin and may indicate that the Dahomey population conserved its purity through the past decades. However, the European climate is different from the one encountered in Benin, i.e., less heat stress, but varying temperatures between winter and summer, and also varying sunlight duration. In addition, the new production environment of the Dahomey cattle implies the reduction of disease infections with potentially improved feeding and housing systems in opposition to the harsh production conditions in Benin (characterized by food and water scarcity and the risk for disease infections). Consequently, this geographical isolation may have altered frequencies for alleles and haplotypes associated with specific genetic features in the Dahomey population.

The livestock production systems in Benin like in the other African countries have experienced a drastic transformation in the last decades (Mwai et al., 2015; Houessou et al., 2019a). New challenges arise in African pastoral regions along with the increasing demand for animal products, food insecurity, and poverty. Indeed, demographic explosion increases the pressures on animal product markets in West Africa and accentuates the need to develop more productive breeds. Simultaneously, anthropogenic activities such as deforestation and urbanization associated with climate changes have shrunk feed and water resources and increased disease challenges, forcing herders to develop new breeding strategies such as herd mobility or feed supplementation (Houessou et al., 2019a; Ahozonlin and Dossa, 2020). In this context, locally adapted animals are required to cope with the various instabilities in production environments, but the indigenous Shorthorn cattle in Benin are increasingly threatened. The trypanotolerant taurine cattle (without any Zebu ancestry) reported in the region by MacHugh et al. (1997) and Hanotte et al. (2002) are progressively replaced by crossbreeds and trypanosusceptible Zebu cattle, including White Fulani, Sokoto Gudali, and Red Bororo (Dossa and Vanvanhossou, 2016; Houessou et al., 2019b; Ahozonlin and Dossa, 2020). In consequence, significant adoption of prophylactic measures is observed in Beninese herds dominated by crossbreed and Zebu cattle in comparison to other local herds (Houessou et al., 2020).

In regard to the increasing uncontrolled crossbreeding, two other indigenous hybrid cattle, the Borgou and Pabli, are also endangered in Benin. The Pabli originally reported in the region of Kerou (Northwest Benin) is scarcely described and is considered as extinct by absorption from crossbreeding with Borgou (Belemsaga et al., 2005; Egito et al., 2007). However, recent evaluations revealed the existence of a population with slight genetic differences in comparison to the Borgou (Scheper et al., 2020). The Borgou cattle mainly located in the Nord-Eastern and Central regions of Benin were described as an intermediate crossbreed between taurine and indicine cattle (Porter, 1991). The origin of Borgou is still under discussion, but it is assumed to be a product of an admixture between the taurine Somba and the White Fulani Zebu (Porter, 1991; Belemsaga et al., 2005). Flori et al. (2014) characterized the admixture in Borgou as an efficient short-term adaptation strategy to environmental conditions and disease pressures and identified different genomic regions involved in adaptive mechanisms. However, the Borgou cattle population is now highly affected by Zebu cattle influence due to admixture (Scheper et al., 2020). The increased crossbreeding with trypanosusceptible Zebu cattle over a short period questions adaptive features such as resistance to diseases in Borgou, Somba, and Lagune taurine populations. It is therefore urgent to gain more insight into the genetic composition of the current Beninese cattle population to ensure the sustainability of livestock production.

To understand the genetic architecture underlying adaptive and productive abilities of various breeds evolving in challenging environments, selection signature analyses have the potential to detect specific genomic footprints in terms of differences in marker allele frequencies or in haplotypic mosaicism (Qanbari and Simianer, 2014; Aliloo et al., 2020; Kim et al., 2020). According to Freedman et al. (2016), admixture and subsequent recombination break down parental haplotypes and expand mosaic regions through the genome. Thus, extensive admixture in local breeds may reduce the signal of strong homozygosity of extended haplotypes involved in adaptive processes. Moreover, the assessment of homozygous-by-descent (HBD) segments or runs of homozygosity (ROH) is valuable to describe a population and investigate demographic histories. HBD are chromosome segments inherited from an ancestor and may be exploited to estimate inbreeding (Leutenegger et al., 2003).

In this study, we aimed to genetically characterize the current endangered cattle breeds in Benin and evaluate the effects of admixture and environmental factors related to late changes in production systems. Specifically, we first assessed the admixture level in the different populations and compared them with historical samples. Second, through selection signature analysis, we investigated genomic regions and biological mechanisms involved or affected by recent natural or artificial selection and admixture in the Beninese cattle breeds. Subsequently, we investigated the genetic differentiation resulting from the isolation of the Dahomey cattle.

## Materials and Methods

### Sampling Design and Genotype Data

**FIGURE 1:**
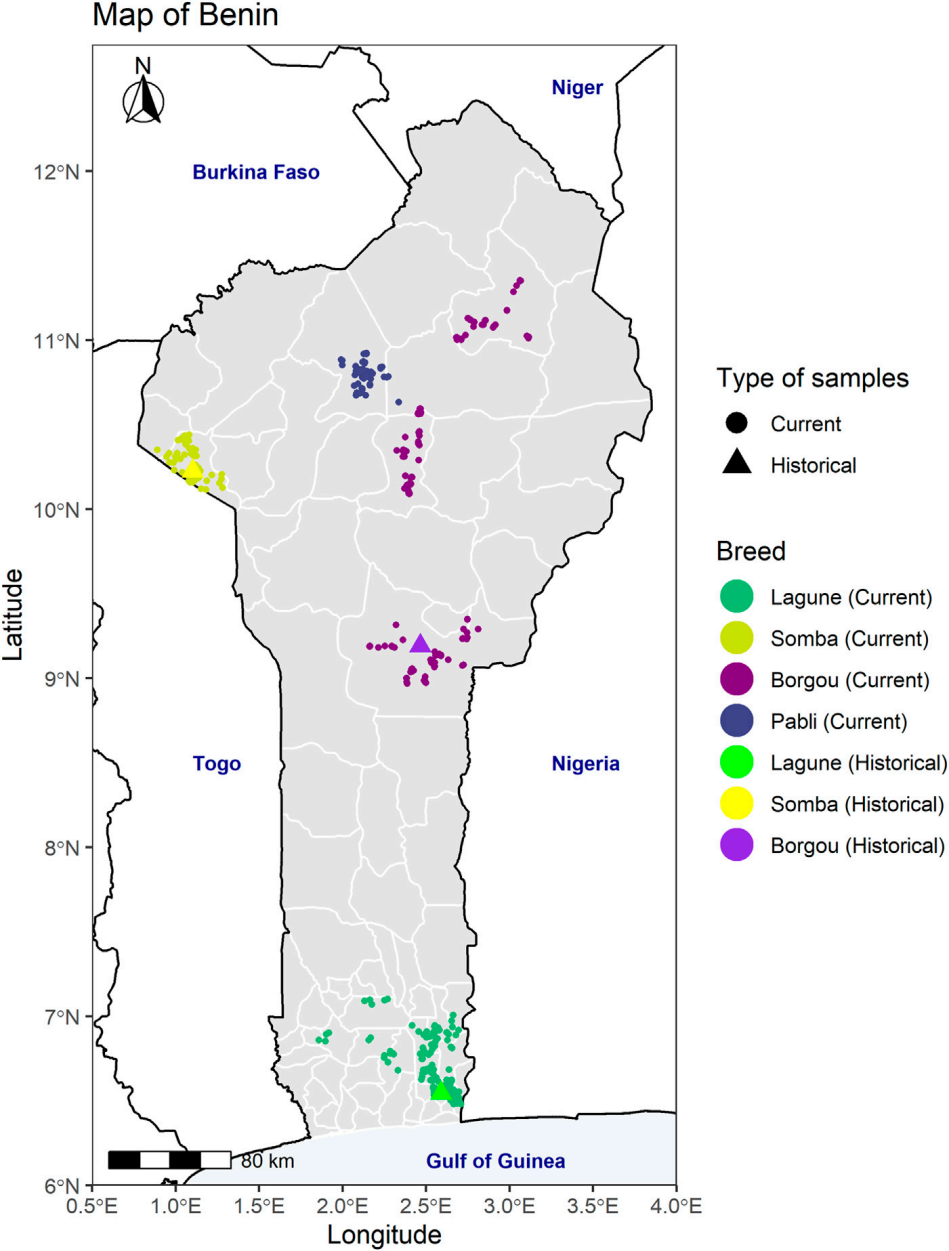
Map of Benin with the sampling locations of the different Beninese cattle populations investigated in the country. For the historical data, the geographical coordinates of each sample are not available. In consequence, the coordinates of the sampling regions as described by Moazami Goudarzi et al. (2001) were plotted: Porto-Novo for the Lagune, Boukombé for the Somba, and the department of Borgou for the Borgou samples.

Hair samples were collected from 449 animals from the four local breeds (Borgou 181, Pabli-Kerou 58, Lagune 150, and Somba 60) in Beninese local farms in 2016 and 2017. The sampling locations were identified ensuring a large coverage of the main geographical distribution of the breeds in the country (Figure 1). In each herd, one animal representing a (pure) local breed was identified by the farmer and selected. To reduce the relatedness between the samples, one or two animals per village were sampled in 90% of the 298 investigated villages. In the remaining villages, additional animals (three to ten animals in total per village) were sampled for the assessment of further socioeconomic and ecological factors including transhumance and climate (see Supplementary Table S1A and Scheper et al. (2020) for more details). Furthermore, thirty Dahomey cattle were sampled in 2019 in 30 different herds across Europe (Germany 23, Austria 4, Switzerland 2, and Czech Republic 1).

The sampled animals were genotyped with the Illumina BovineSNP50 BeadChip. SNP variants with a call rate <95% and a minor allele frequency (MAF < 0.01) as well as animals with a high percentage of missing genotypes (call rate < 90%) were filtered out in PLINK (Purcell et al., 2007). The final genotype dataset including 40,109 SNP from 460 cattle was used for different analyses.

### Description of the Current Beninese Cattle Population

#### Population Structure

The genetic structure of our samples was first assessed by applying the fastStructure software (Raj et al., 2014). The algorithm was run using the simple prior model and testing different numbers of populations (K) ranging from 2 to 10. Afterward, the optimal K was defined using the chooseK.py function. The identified optimal K was finally incorporated into the logistic prior model, to estimate individual genetic proportions describing the population structure, following the recommendations by Raj et al. (2014).

Second, we applied the unsupervised k-means clustering in the “adegenet” R package (Jombart, 2008; Jombart and Ahmed, 2011) to define homogeneous genetic clusters and to exclude potential outgroups animals from the breeds due to sampling. The find.clusters function was applied, considering the following parameters: maximum number of clusters (max.n.clust) = 100, number of principal components (n.pca) = 100, number of iterations in each run (n.iter) = 10^9^, number of starting centroids in each run (n.start) = 30, and the default parameters for the remaining arguments (see package description for further details, Jombart, 2008; Jombart and Ahmed, 2011). Finally, the optimal number of genetic clusters was defined by choosing the k-value based on the Bayesian information criterion (BIC), as recommended by the authors (Jombart 2008; Jombart and Ahmed 2011).

#### Genome-Wide Inbreeding Coefficients

We utilized the “RzooRoH” package (Druet and Gautier, 2017; Bertrand et al., 2019) in R to identify HBD and infer the contribution of ancestors from different past generations to inbreeding in our current cattle populations. The software applies a hidden Markov model (HMM) to fit the individual genome as a succession of HBD and non-HBD segments, considering marker allele frequencies, genotyping error rates, and intermarker genetic distances (Druet et al., 2020). In a multiple HBD class model, HBD segments are assigned to K age-related classes associated with different rates (R_k_). The class rates (R_k_) are related to an expected length and exponential distribution of HBD segments. They are equivalent to twice the number of generations to the common ancestor. The proportion of the genome belonging to a specific HBD class is defined as “realized autozygosity” in the respective HBD class. Similarly, the genome-wide inbreeding coefficient is estimated as the cumulative fraction of the genome that is HBD in the current population with respect to an ancestral base population. As suggested by the authors of the “RzooRoH” package (Druet and Gautier, 2017; Bertrand et al., 2019), we applied a default “MixKR” model with 10 classes (9 HBD classes and 1 non-HBD class) and predefined rates (R_k_) 2, 4, 8, 16, 32, 64, 128, 256, 512, and 512, respectively.

### Comparison of Current Beninese Cattle Population With Historical Data

#### Extension of the Genotype Dataset With Historical Data

We contrast the genotype of the current Beninese cattle population with available historical genotypes, retrieved from the WIDDE database (Sempéré et al., 2015). In a first step, the genotype data of 133 animals from Beninese cattle breeds (Borgou 45, Lagune 44, and Somba 44) sampled in different locations (see Figure 1 and Supplementary Table S1B) between 1997 and 2000 (Moazami Goudarzi et al., 2001; Gautier et al., 2009) were merged to the genotype data of our new samples. The dataset with 42,802 SNP from 588 animals was submitted to quality control in PLINK (Purcell et al., 2007), with the parameters --geno 0.05, --mind 0.25, and --maf 0.01. After quality control, the final genotype dataset including 32,533 SNP from 586 cattle was used to apply principal component analysis and unsupervised k-means clustering. Second, further African Shorthorn taurine breed (Baoulé), African Longhorn taurine breed (N’Dama), African indicine breeds (Zebu White Fulani and Zebu Bororo), Asian indicine breeds (Gir, Brahman, Ongole, and Nellore), EUT breeds (Angus, Holstein, Charolais, Shorthorn, and Salers), and African hybrid (Kuri) were included. This extension resulted in 52,341 SNP from 997 animals. However, only 30,637 SNP from 997 animals passed the quality control procedure (with the parameters --geno 0.05, --mind 0.25, and --maf 0.01 in PLINK) and were considered in the principal component analysis, unsupervised k-means clustering, and admixture tests (see below for more details). Finally, 21 Gayal (*Bos frontalis*, Gao et al., 2017) samples were added to the previously extended dataset for the estimation of admixture proportion through the calculation of the f_4_-ratio. Likewise, we used the same filtering parameters (--geno 0.05, --mind 0.25, and --maf 0.01) in PLINK. The genotype data consisted of 52,364 SNP and 30,228 SNP from 1,018 animals before and after quality control, respectively. All the genotype data exploited in this study are fully described and available in a public repository (see the section “*Data Availability Statement*”). Before being merged with our samples, the historical genotype data were remapped with the current reference assembly ARS-UCD1.2/bosTau9 (GenBank Bioproject PRJNA391427) and flipped (with the --flip flag in PLINK) to correct for strand inconsistency (Purcell et al., 2007).

#### Principal Component Analyses and Clustering

The genetic divergence between the Beninese cattle populations and other AFT was assessed using the principal component analysis (PCA) in PLINK (Purcell et al., 2007) and using the unsupervised k-means clustering in “adegenet” (Jombart, 2008; Jombart and Ahmed, 2011) in R. The analyses were subsequently repeated considering EUT and indicine breeds in order to investigate potential introgression of these breeds in our samples, especially in Dahomey. The optimal number of genetic clusters for the unsupervised k-means clustering was defined using the same approach as described above.

#### Admixture and Estimation of Ancestral Proportion

We tested admixture and inferred admixture proportion in the current and historical Beninese indigenous cattle populations by means of the three-population test (F_3_) and the F_4_-ratio estimation in Admixtools, respectively (Patterson et al., 2012). The methods are based on f-statistics, corresponding to the average of F values over markers. The Admixtools software uses allele frequencies of the available samples to estimate unbiased f_3_ and f_4_ statistics (see Patterson et al., 2012, for more details). The three-population test is a formal test of admixture. Negative f_3_ (X; B, C) indicates that the allele frequencies in population X tend to be intermediate between B and C and indicates admixture in X populations from populations related to B and C. The f_4_ statistics were used to infer admixture proportions in the Beninese cattle populations (target populations) through the calculation of the F_4_-ratio or alpha (Eq. 3; Patterson et al., 2012). We considered the phylogeny model introduced by Flori et al. (2014) to estimate alpha, as AFT ancestral proportions in our target populations.

$$\text{Alpha}=\frac{{\text{f}}_{4}\left(\text{A},\text{O};\text{B},\text{C}\right)}{{\text{f}}_{4}\left(\text{A},\text{O};\text{X},\text{C}\right)},$$ (3)

where X is the target population, A is the Salers population as EUT ancestral, O is the Gayal as outgroup population, B is Baoulé (BAO) as the Shorthorn AFT reference population, and C is Gir as the indicine reference population.

#### Detection of Regions Under Recent Selection—Gene Annotation and QTL Mapping

We investigated divergence in extended haplotype homozygosity between the current and historical Beninese cattle populations, in order to detect positive selection signatures or genomic footprints left by recent demographic events. Specifically, we compared DAH_cur, LA_cur, and LA_out with LA_hist; SO_cur with SO_hist; and BO_cur and Adm_cur with BO_hist. The cross-population–extended haplotype homozygosity (XP-EHH) approach (Sabeti et al., 2002) was implemented in the “Rehh” package in R (version 3.1.2; Gautier and Vitalis, 2012; Gautier et al., 2017). Prior to the analyses, the main genotype was phased and missing variants were imputed in Beagle 5.1 (Browning et al., 2018). Afterward, integrated site-specific extended haplotype homozygosity (iES) was calculated for each focal marker in the respective population with the “Rehh” package. The XP-EHH statistics were computed as the standardized log ratio of the iES of the two populations. One-sided *p*-values were estimated for XP-EHH to identify strong extended homozygosity in our current populations relative to the respective historical populations (as described above). The estimated *p*-values were subsequently adjusted (Benjamini and Hochberg, 1995) in R. Variants with adjusted *p*-values ≤ 0.05 were considered as significant. In addition, we used a conservative approach similar to those described in previous studies (Flori et al., 2014; Bertolini et al., 2020; Han et al., 2020) and defined as candidate regions under selection, sliding windows of 0.5 MB spanning at least three significant markers. The calc_candidate_regions function in “Rehh” was used to detect the candidate regions. Neighboring windows with significant SNP were merged to one candidate region. The peak of each candidate region, i.e., the SNP with the lowest adjusted *p*-values in a region, was considered as core SNP.

Genes located in the candidate regions for positive selection signatures were annotated from the Ensembl genome database (2020) (http://www.ensembl.org/biomart/martview/) and submitted to gene ontology (GO) enrichment analysis using the Gene Ontology web-tool (Ashburner et al., 2000; Gene Ontology Consortium, 2019). Fisher’s exact threshold of *p*-values < 0.01 was considered to identify overrepresented GO terms for biological processes and Reactome pathways. In addition, QTL that overlapped with the candidate regions under selection were mapped from the online data analysis tools of the cattle database (Hu et al., 2019) and summarized in major production and functional categories: milk, carcass quality, reproduction, body weight, conformation, feed intake, heat tolerance, and health traits (see Supplementary Table S2 for more details). Subsequently, for each population, we calculated the frequency of the QTL which is equal to the number of candidate regions overlapping with the given QTL per the total number of candidate regions.

## Results

### Population Structure and Inbreeding in the Current Beninese Cattle Population

#### Structure of the Current Population

**FIGURE 2:**
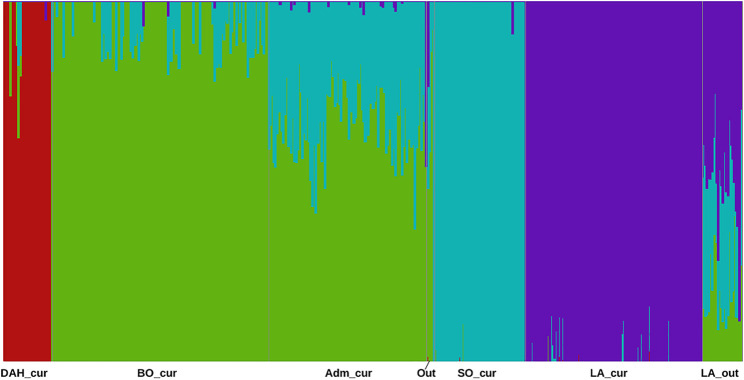
Barplot for admixture proportions inferred by fastStructure in the Beninese cattle populations. Dah_cur = current Dahomey; LA_cur = current pure Lagune; LA_out = admixed Lagune; SO_cur = current Somba; BO_cur = current Borgou highly admixed; Adm_cur = current moderately admixed animal including Pabli with some Borgou, Out = Outgroups (see Table 1 for more information).

The analyses with the fastStructure algorithm revealed four model components to explain the population structure of our samples. Similarly, the model complexity that maximizes marginal likelihood was equal to four. Considering the posterior mean of admixture proportion in the logistic model, we identified three components representing Somba, Lagune, and Dahomey samples, respectively (Supplementary Table S3). The fourth component was mainly made based on Borgou animals. However, some hybrid samples displayed genetic proportions across two or more components. This structure was similar to those obtained with adegenet (Figure 2). The unsupervised k-means clustering presented an optimal K (number of clusters, Supplementary Figure S1) equal to 6 and displayed the hybrid animals in different three clusters (Supplementary Figure S1 and Table 1). Consequently, a large Lagune group named LA_cur (*n* = 110) was separated from other Lagune samples called LA_out (*n* = 25). Forty-two Borgou and 56 Pabli animals (except two outgroups) formed a homogeneous cluster named Adm_cur (*n* = 98). The remaining Borgou animals were allocated to the BO_cur cluster. The Dahomey and the Somba correspond to the DAH_cur and SO_cur, respectively (Table 1). LA_cur, LA_out, DAH_cur, SO_cur, BO_cur, and Adm_cur were considered as populations for further analyses instead of original breed assignments.

**TABLE 1:** Genetic clusters generated from unsupervised k-means clustering applied on the current Beninese cattle population.

Generated genetic clusters		Animals	Breed name assigned by the sampling				
			Borgou	Dahomey	Lagune	Pabli	Somba
Lagune_current	LA_cur	110			110		
Lagune_outgroup	LA_out	25			25		
Dahomey_ current	DAH_cur	30		30			
Somba_ current	SO_cur	57					57
Borgou_ current	BO_cur	135	135			2^a^	
Undescribed_admix	Adm_cur	98	42		2^a^	56	1^a^
Total		455	177	30	137	58	58

a Five animals that did not show clear adherence to the main groups were considered as outgroups and excluded from the generated genetic clusters.

#### Genomic Inbreeding Coefficients

**FIGURE 3:**
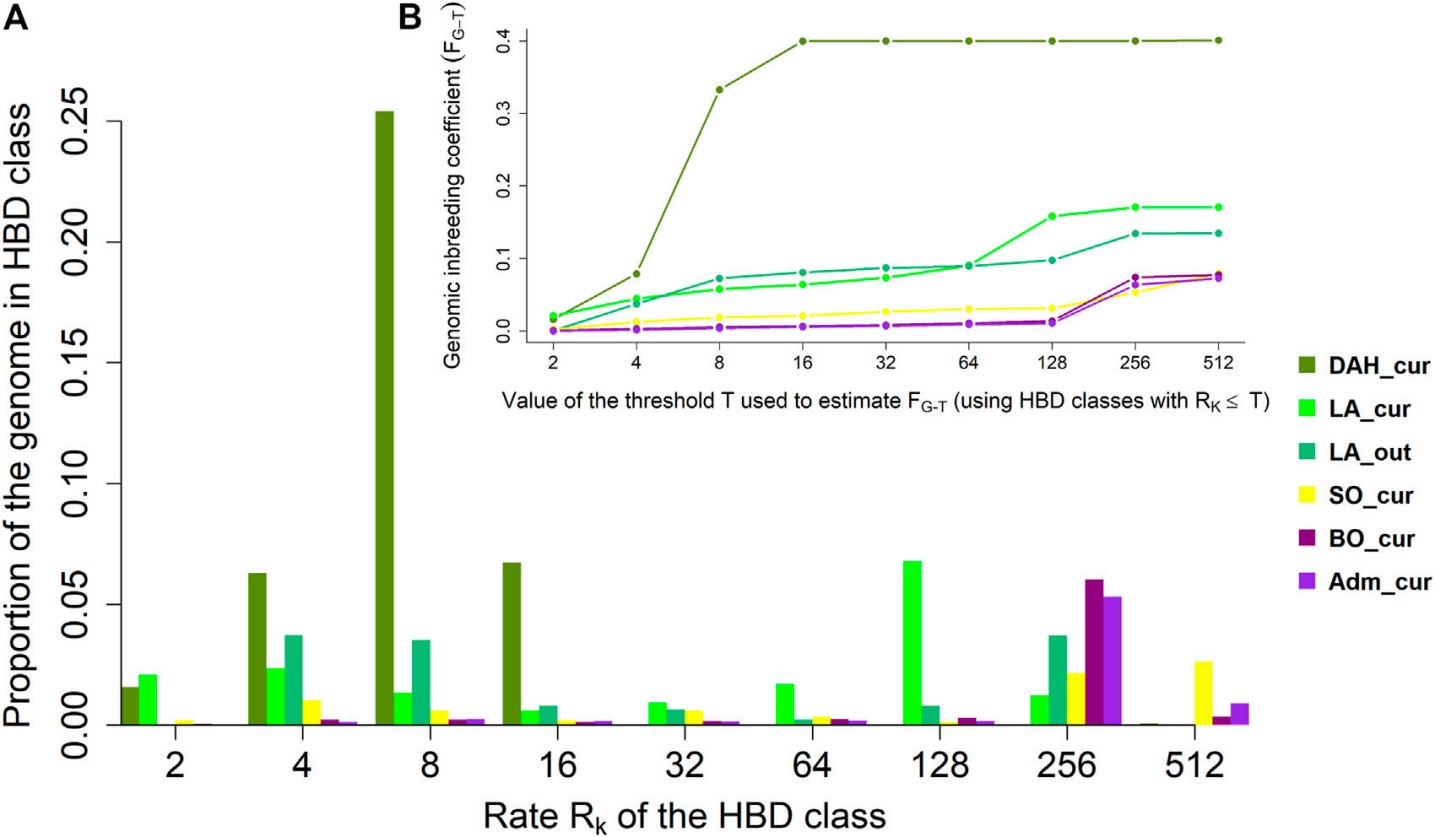
**(A)** Average realized autozygosity and **(B)** cumulative genomic inbreeding associated with different age-related homozygous-by-descent (HBD) classes in Beninese cattle populations. Dah_cur = current Dahomey; LA_cur = current pure Lagune; LA_out = admixed Lagune; SO_cur = current Somba; BO_cur = current Borgou highly admixed; Adm_cur = current moderately admixed animal including Pabli with some Borgou.

The genome-wide contributions and genomic inbreeding coefficients with respect to different age-related HBD classes in the Beninese cattle populations are presented in Figures 3A,B, respectively. Overall, 40% of the genome of DAH_cur samples was HBD and was related only to HBD classes with R_k_ ≤ 16. We observed a major contribution of autozygosity from the HBD class with R_k_ = 8, accounting for 63.32% of the total HBD proportion in DAH_cur. The HBD classes with R_k_ = 4 and R_k_ = 16 contributed to 15.70 and 16.80% of the overall autozygosity, respectively. The estimated genomic inbreeding coefficients were 0.17 and 0.13 in LA_cur and LA_out, respectively, when considering all HBD classes (i.e., the most remote base population). The major contribution of autozygosity in LA_cur came from the ancient HBD class with R_k_ = 128 (40% of the HBD proportion). Recent HBD classes with R_k_ = 4 and R_k_ = 8 explained 13.78 and 7.80% of the total autozygosity in LA_cur, respectively, whereas 27.67 and 26.11% of the overall autozygosity in LA_out were derived from the two classes. Tracing back to the oldest ancestors, the fraction of the genome that was HBD in SO_cur was equal to 0.08 and was mainly originated from very ancient HBD classes with R_k_ = 256 (27% of the total HBD proportion) and R_k_ = 512 (33% of the total HBD proportion). The contribution of recent classes to autozygosity in SO_cur was lower (≤13% of the HBD loci). The estimated genomic inbreeding coefficients were 0.08 for BO_cur and 0.07 for Adm_cur. The HBD class with R_k_ = 256 was the main source of autozygosity, contributing to 77.94 and 73.09% of the total HBD proportions in BO_cur and Adm_cur, respectively.

### Comparison of Current Beninese Cattle Population With Historical Data

#### Principal Component Analyses and Clustering

**FIGURE 4:**
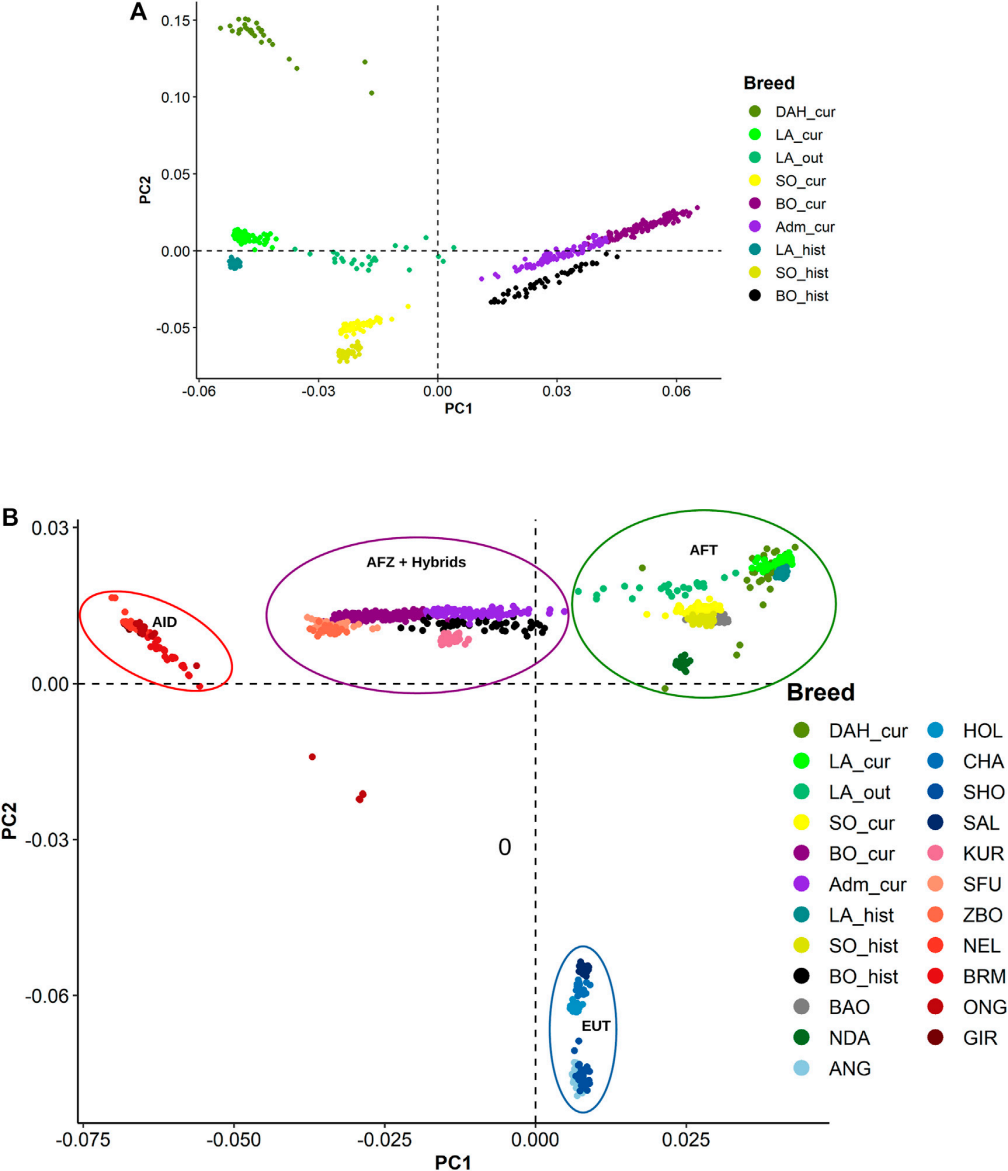
Principal components (PC) displaying genomic divergence: **(A)** in the different Beninese cattle populations; **(B)** in the Beninese cattle with additional African and European taurine (EUT) breeds, African and Asian indicine breeds, and African crossbreeds. Dah_cur = current Dahomey; LA_cur = current pure Lagune; LA_out = admixed Lagune; SO_cur = current Somba; BO_cur = current Borgou highly admixed; Adm_cur = current moderately admixed animal including Pabli with some Borgou; LA_hist = historical Lagune; SO_hist = historical Somba; BO_hist = historical Borgou; ANG = Angus; HOL = Holstein; CHA = Charolais; SHO = Shorthorn; BAO = Baoulé; NDA = N’Dama; GIR = Gir; SFU = Sudanese Fulani; ZBO = Zebu Bororo; BRM = Brahman; ONG = Ongole; NEL = Nellore; KUR = Kuri; SAL = Salers.

The first and second principal components (PC) from PCA presented a clear separation of the Beninese breeds (Figure 4A). Within each breed, recent and historical samples were distinctly displayed along the second PC. The Dahomey cattle (DAH_cur) were projected next to LA_cur on PC1 but have a major contribution to PC2. Similar differentiations between current and historical samples were observed with the unsupervised k-means clustering, especially in Borgou and Lagune (BIC values suggested seven clusters, see Supplementary Figure S2A). Indeed, the unsupervised k-means clustering assigned the animals from Adm_cur, BO_cur, and BO_hist in three different clusters (clusters 2, 6, and 7), respectively (Supplementary Table S4A). Similarly, the current Lagune samples (LA_cur and LA_out) were grouped into cluster 4 and historical samples (LA_hist) into cluster 1. DAH_cur formed its own cluster (cluster 4). However, the current and historical Somba samples (SO_cur and SO_hist) were jointly grouped into cluster 5.

We repeated the diversity analyses on the extended dataset including additional AFT, EUT, AFZ, Asian indicine (AID), and hybrids. Considering the PCA, the taurine animals (AFT and EUT) were separated from indicine (AFZ and AID) and hybrids samples along the first component, whereas the second component (PC2) showed a separation between the AFT and EUT (Figure 4B). Interestingly, the Dahomey was aggregated with the Lagune far away from EUT. The AID (Gir, Brahman, Ongole, and Nellore) also were clearly separated from the AFZ (ZFU and ZBO) and hybrids. Eight clusters (low BIC at k = 8; Supplementary Figure S2B) were identified from the unsupervised k-means clustering. Current and historical Somba samples (SO_cur and SO_hist) formed one genetic group (cluster 2) with the other AFT (BAO and NDA). The Lagune samples (LA_cur, LA_out, and LA_hist) were grouped into cluster 2. Similarly, DAH_cur is displayed in one cluster (cluster 1). Moreover, the hybrids (Adm_cur, BO_cur, and BO_hist) were grouped with Kuri, ZFU, and ZBO in cluster 6. The other genetic groups consisted of the EUT and AID (Supplementary Table S4B).

#### Formal Test of Admixture and Inference of Ancestral Proportion

The formal test of admixture of three populations resulted in positive f_3_-ratios for DAH_cur, LA_cur, and SO_cur (Table 2). In contrast, we obtained negative statistics for LA_out, BO_cur and Adm_cur with the most significantly negative f_3_ value for BO_cur (f_3_ = −0.08; Z = −39.08). Considering the historical data, the f_3_-ratio test resulted in positive values for LA_hist but was negative for SO_hist and BO_hist.

**TABLE 2:** f_3_ and f_4_ statistics for formal test of admixture in Beninese cattle populations.

Target^a^	f_3_-ratio	z-score	Alpha^b^
DAH_cur	0.376	26.927	0.974
LA_cur	0.111	35.612	1.000
LA_out	−0.021	−9.677	0.872
SO_cur	0.005	2.492	0.963
BO_cur	−0.083	−39.083	0.474
Adm_cur	−0.048	−28.460	0.615
LA_hist	0.110	35.501	1.008
SO_hist	−0.006	−4.542	0.972
BO_hist	−0.098	−55.732	0.626

a Dah_cur = current Dahomey; LA_cur = current pure Lagune; LA_out = admixed Lagune; SO_cur = current Somba; BO_cur = current Borgou highly admixed; Adm_cur = current moderately admixed animal including Pabli with some Borgou; LA_hist = historical Lagune; SO_hist = historical Somba; BO_hist = historical Borgou.

b Alpha values represent the estimates of the proportion of AFT in the different populations.

The estimation of the ancestral AFT with the f_4_-ratio test revealed high proportions (alpha values superior to 0.97) in DAH_cur, LA_cur, and SO_cur, respectively (Table 2). In contrast, lower alpha values were found for LA_out, Adm_cur, and BO_cur. In comparison to the historical samples, we observed slight reductions of AFT ancestral proportions in Somba (0.96 for SO_cur against 0.97 for SO_hist) and in Adm_cur (0.62 against 0.63 for BO_hist). The current Borgou (BO_cur) displayed an important reduction of AFT ancestral proportions (alpha = 0.47) in comparison to historical samples (BO_hist). For LA_cur and LA_hist, the estimated AFT ancestral proportions were equal to 1.00, respectively.

#### Selection Signatures

##### Dahomey and Lagune

**FIGURE 5:**
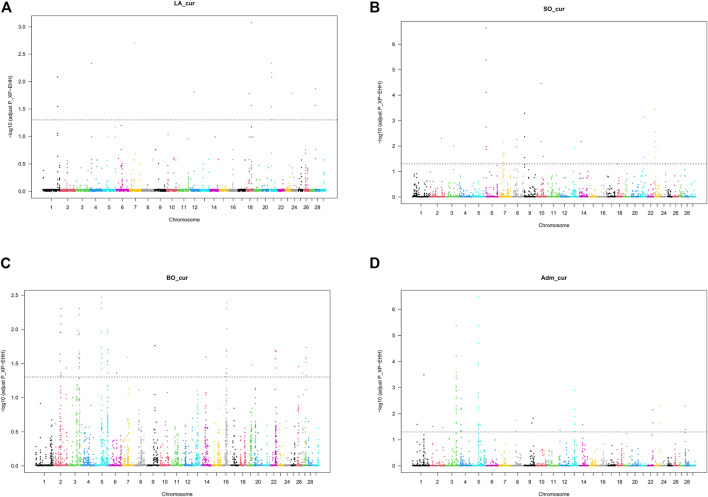
Manhattan plot for cross-population extended haplotype homozygosity (XP-EHH) test for selection signature in Beninese cattle populations: **(A)** LA_cur, current Lagune pure population, **(B)** SO_cur, current Somba population, **(C)** BO_cur, current Borgou highly admixed, and **(D)** Adm_cur, moderately admixed hybrids.

**FIGURE 6:**
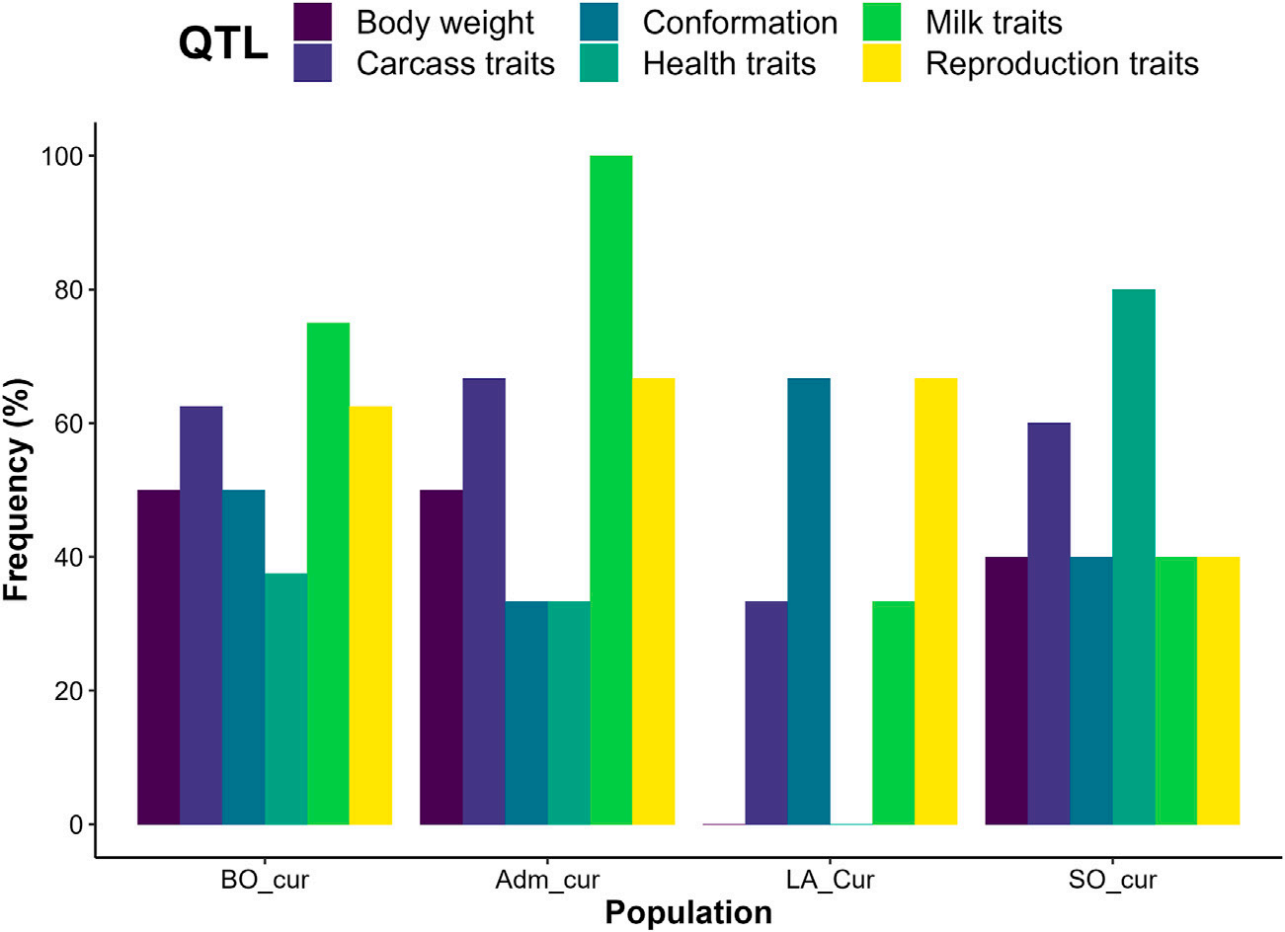
Proportion of candidate regions with quantitative trait loci (QTL) controlling varied trait categories. LA_cur = current pure Lagune; SO_cur = current Somba; BO_cur = current Borgou highly admixed; Adm_cur = current moderately admixed animal including Pabli with some Borgou.

We detected no significant SNP presenting strong extended homozygosity in DAH_cur and LA_out relative to LA_hist. In contrast, the current Lagune population (LA_cur) displayed 19 significant SNP for positive selection (Figure 5A and Table 3). Among the SNP, 12 were positioned in a total of three candidate regions on BTA1, 18, and 21. The region 15.5–16 Mb on BTA21 contained the largest number of significant SNP (six SNP). The region 47.5–48 Mb on BTA18 included the most significant SNP rs110495745 (*p_adjust* = 8.40 × 10^−04^), which is positioned within the *WDR87* gene. In total, 9 candidate genes were annotated within the three regions. GO enrichment analysis identified functional enriched terms such as cellular processes (Table 4). QTL associated with reproduction (67%) and conformation (67%) were predominant in the candidate regions (Figure 6).

**TABLE 3:** Candidate regions harboring positive recent selection signatures in Beninese cattle populations.

Population^a^	BTA	Region (Mb)		Number of sign. SNP	Adjust p-value		Genes^b^
		Start	End		Min	Max	
LA_cur	1	137.5	138	3	0.008	0.029	
	18	47.5	48	3	0.001	0.027	***WDR87*** *, ZNF345, ZFP30*
	21	15.5	16	6	0.005	0.049	*SV2B*
SO_cur	6	4	4.5	6	0.000	0.013	QRFPR
	7	45	45.5	5	0.018	0.049	*FSTL4*
	9	4	4.5	3	0.001	0.028	
	23	7	7.5	3	0.000	0.007	***BOLA-DMA***, ***BOLA-DMB***, ***BRD2***, *PSMB8*
	23	11.5	12	3	0.011	0.028	*MDGA1, ZFAND3*
BO_cur	2	70	71	9	0.005	0.050	EN1, MARCO
	3	86	86.5	3	0.010	0.026	HOOK1
	3	100	100.5	5	0.006	0.035	**MAST2**, RAD54L, POMGNT1
	3	101	101.5	4	0.005	0.027	HECTD3, KIF2C
	5	54.5	55.5	8	0.003	0.031	*LRIG3*
	5	111	111.5	6	0.010	0.033	GRAP2, **ENTHD1**, FAM83F
	16	46.5	47	6	0.004	0.025	***DNAJC11*** *,* RNF207, *PLEKHG5, THAP3*
	27	34	34.5	3	0.026	0.047	*PLEKHA2, ADAM32,*
Adm_cur	3	100	100.5	6	0.001	0.007	*MAST2, RAD54L, POMGNT1*
	3	101	102	13	0.000	0.023	HECTD3, **RNF220**, **IPP**
	4	20	21	6	0.003	0.049	TMEM106B, SCIN, ARL4A
	5	54.5	55.5	8	0.000	0.025	LRIG3
	5	57	57.5	6	0.000	0.040	ANKRD52, SLC39A5, RNF41, DNAJC14
	13	45.5	46	3	0.001	0.047	—

a LA_cur = current pure Lagune; SO_cur = current Somba; BO_cur = current Borgou highly admixed; Adm_cur = current moderately admixed animal including Pabli with some Borgou.

b Genes harboring the core SNP are displayed in bold. The complete list of the genes located in the candidate regions are presented in Supplementary Table S5.

**TABLE 4:** Enriched gene ontology (GO) biological process for genes in candidate regions under positive selection in Beninese cattle populations.

Population^a^	GO biological process	p- value	N^b^	Genes
LA_cur	Cellular process (GO:0009987)	5.94E-03	2	*SV2B, ENSBTAG00000054913*
SO_cur	Antigen processing and presentation (GO:0019882)	5.44E-19	11	*BOLA-DOA, BOLA-DMA, TAP1, PSMB8, TAPBP, DSB, BOLA-DMB, BOLA-DYA, BOLA-DOB, BOLA-DMA, BOLA-DIB*
	Adaptive immune response (GO:0002250)	2.74E-11	9	*BOLA-DOA, BOLA-DMA, TAP1, DSB, BOLA-DMB, BOLA-DYA, BOLA-DOB, BOLA-DMA, LOC618733*
	MHC protein complex assembly (GO:0002396)	3.98E-05	2	*BOLA-DMA, TAPBP*
	Cellular response to steroid hormone stimulus (GO:0071383)	6.61E-03	2	*RXRB, DAXX*
	Proteasomal ubiquitin-independent protein catabolic process (GO:0010499)	7.03E-04	2	*PSMB8, PSMB9*
	DNA conformation change (GO:0071103)	9.03E-03	3	*H2B, BRD2, DAXX*
BO_cur	GO:000700: inner mitochondrial membrane organization (GO:0048519)	2.42E-03	2	*TAZ, DNAJC11*
	Negative regulation of biological process (GO:0048519)	5.20E-03	2	
	Cytoplasmic microtubule organization (GO:0031122)	7.61E-03	2	*HOOK1, PLK3*
	Biological regulation (GO:0065007)	6.25E-03	16	*EIF2B3, KLHL21, ADAM9, KIF2C, TNFRSF25, MAST2, THAP3, PTCH2, ZBTB48, HES2, PLK3, TAS1R1, PLEKHG5, EN1, GPBP1L1, FAM83F*
Adm_cur	Pigment biosynthetic process	7.01E-03	2	*UROD, PMEL*
	ATP-dependent chromatin remodeling	7.90E-03	2	*DMAP1, SMARCC2*
	Response to chemical	9.33E-03	3	*SLC39A5, PLK3, ENSBTAG00000051912 (taste receptor type 2)*
	G1/S transition of mitotic cell cycle	9.50E-03	2	*CDK2, PLK3*

a LA_cur = current pure Lagune; SO_cur = current Somba; BO_cur = current Borgou highly admixed; Adm_cur = current moderately admixed animal including Pabli with some Borgou.

b Number of the identified genes.

##### Somba

The analysis of positive selection signatures in SO_cur relative to SO_hist detected 35 significant SNP (Figure 5B). Five candidate regions under recent selection in SO_cur were identified on BTA6, 7, 9, and 23, harboring in total 20 significant SNP. The region 4–4.5 Mb on BTA6 displayed the majority of the significant SNP (6 SNP) and the most significant SNP (rs42405104, *p_adjust* = 2.34 × 10^−07^). This SNP is not positioned in any gene. However, the second most significant SNP (*p_adjust* = 3.57 × 10^−04^) is positioned in the vicinity of three genes (*BOLA-DMB*, *BOLA-DMA* and *BRD2*) on BTA 23. In total, 33 genes were mapped in the candidate regions. GO enrichment analysis of the genes revealed highly significant GO biological processes such as antigen processing and adaptive immune response (Table 4). We observed that the majority of the candidate regions under positive selection in SO_cur overlapped with QTL affecting heath traits (80%) and carcass quality (60%, Figure 6).

##### Crossbreeds

We detected several genomic regions under recent selection in BO_cur and Adm_cur relative to BO_hist (Figures 5C,D). A total of 77 SNP displayed strong homozygosity in BO_cur, with adjusted *p_values* for XP-EHH below the significance threshold (*p_adjust* ≤ 0.05). Among these SNP, 44 were located in eight candidate regions on BTA2, 3, 5, 16, and 27 (Table 3). The regions that spanned from 70 to 71 Mb on BTA2 and from 54.5 to 55.5 Mb on BTA5 contained the largest number of significant SNP (9 and 8 significant SNP, respectively). The later region hosted the highest peak with the SNP rs41637710 (*p_adjust = 0.003*). This SNP was not in the vicinity of any gene. However, three significant SNP (rs110903828 on BTA27, rs41637109 on BTA16, and rs43361717 on BTA3) were positioned within the genes *HTRA4*, *DNAJC11*, and *TMEM53*, respectively. A total of 49 genes were mapped within the genomic candidate regions. GO enrichment analysis revealed several significant GO biological processes including cytoplasmic microtubule organization and inner mitochondrial membrane organization (Table 4). Moreover, several QTL affecting animal performances overlapped with the regions under positive selection in BO_cur. QTL associated with milk (75%), carcass (63%), and reproduction traits (63%) were the most represented (Figure 6).

In Adm_cur, we identified 69 significant SNP under positive selection. A subset of 42 SNP was located in six candidate regions on BTA3, 4, 5, and 13. Three candidate regions in Adm_cur overlapped with those detected in BO_cur (Table 3). Similarly, the SNP rs41637710 located on BTA5 (54.5–55.5 Mb) showed the lowest adjusted *p*-value (*p_adjust* = 3.38 × 10^−07^). The segment on BTA3 (101–102 Mb) hosted the largest number of extreme SNP (13 SNP). The candidate regions in Adm_cur included 63 genes. The genes *DNAJC11*, *ENTHD1*, and *MAST2* hosted significant SNP, whereas others (*IL23A*, *PAN2*, and *CNPY2*) were mapped at a close distance to the significant SNP. GO enriched terms included biological processes such as pigment biosynthetic process and response to chemicals (Table 4). Like in BO_cur, the majority of QTL, located in the candidate regions of positive selection in Adm_cur, were related to milk (100%), carcass (67%), and reproduction traits (67%, Figure 6).

## Discussion

### Population Structure and Admixture Tests

The results of the population structure analyses are in line with the breed foundation of the different cattle populations in Benin and their divergence from other African and European cattle breeds (Mwai et al., 2015). First, we observed high proximity of the Somba and Baoulé populations as Savannah Shorthorns and their separation to the Dwarf (forest) Lagune Shorthorn (Rege et al., 1994). In addition, the divergence of the Shorthorn breeds from the Longhorn N’Dama illustrates the rich genetic diversity of West African indigenous cattle breeds and the necessity to unravel specific signatures in each population. Second, our study confirms the Lagune origin of the Dahomey cattle. We found neither indicine nor EUT background in the Dahomey population. The genetic purity of the Dahomey cattle is probably due to their promotion in a close production system organized by the Dahomey-Zwergrind breeder association aiming at the conservation of the breed (http://www.dahomey-zwergrind.com). The inference of the oldest common ancestor dated from eight generations ago (approximately 24 years ago). The high genomic inbreeding coefficient in R_k_ = 8 (4 generations equal to 16 years ago) in the Dahomey population may be related to the recent creation of the association in 2001. These results suggest that the founders of the current Dahomey population kept by farmers may have originated from a small number of Dahomey cattle four generations ago (http://www.dahomey-zwergrind.com). However, the low frequency of short ROH segments, resulting in low genomic inbreeding coefficients (<0.1) in very young classes (R_k_ ≤ 4), indicates a reduction of mating between related individuals in recent generations (Druet and Gautier, 2017). Exchange of breeding animals between the association members and consideration of Dahomey cattle currently held in different zoos across Germany and other European countries (Zootierliste, 2020: https://www.zootierliste.de) may contribute to control inbreeding and to increase genetic diversity in the population currently managed by the Dahomey–Zwergrind breeder association.

The results of the PCA analyses differentiated historical and current populations as well as populations affected by admixture. The identification of admixed animals (LA_out) from relatively pure Lagune is confirmed by the formal admixture test, the three-population test, and the estimation of admixture proportions. Our findings are in accordance with the increasing crossbreeding in Lagune cattle due to the extension of transhumance as reported by Scheper et al. (2020) and Ahozonlin and Dossa (2020). The large genomic inbreeding in the Lagune (LA_cur) is in line with the small number of the original populations formerly distributed in clusters across West African coastal and forest regions (Rege, 1999). Fortunately, the estimation of high AFT ancestral proportion in LA_cur suggests the existence of a relatively pure population, which may be valuable for the conservation of this indigenous taurine breed. The estimated alpha value of 1.00 may be related to the reference population considered. However, the Baoulé (BAO) is the closest shorthorn taurine with available historical genotype data, whereas the GIR is the purest indicine reference population as the majority of AFZ are admixed (Flori et al., 2014).

We found that Somba cattle are less affected than Lagune by Zebu introgression. Previous studies observed that its habitat in the hilly region of Atacora protected from Zebu introgression (Rege et al., 1994; Hall et al., 1995). In comparison to the location of the other local breeds (Northeastern and Southern Benin), the lower pressure of transhumance in Boukombe and lower economic and demographic pressures, resulting in less “modernization” of cattle management and crossbreeding, are some advantages (Houessou et al., 2019a; Scheper et al., 2020). However, the lower AFT ancestral proportion in SO_cur compared to SO_hist confirms the threat of admixture in Somba cattle mainly caused by entrustment practices (Hall et al., 1995; Dossa and Vanvanhossou, 2016; Vanvanhossou et al., 2021). In addition, the negative and positive f-statistics in the historical (SO_hist) and the current (SO_cur) Somba populations, respectively, indicate former introgression episodes followed by genetic drift (Patterson et al., 2012; Kim et al., 2020). We also observed that Somba cattle remain less inbred despite the reduction of population size and their shrinkage into the unique location of Boukombe (Dossa and Vanvanhossou, 2016).

The results from fastStructure corroborate the Somba background of the Beninese crossbreeds. In addition, the hybrid populations (BO_cur and Adm_cur) presented a relatively low genetic proximity to the AID (Gir, Brahman, Ongole, and Nellore), but they were clustered close to the AFZ (ZFU and ZBO). These results are in agreement with their origin as described by different authors (Atchy, 1976; Flori et al., 2014). The identification of different levels of admixture in the hybrid samples was confirmed by the f_3_ and f_4_ admixture tests. The lower AFT ancestral proportion in BO_cur compared to the historical population BO_hist confirms the increasing introgression of AFZ in smallholder Borgou herds as indicated by Scheper et al. (2020). BO_cur representing more than 75% of the current Borgou samples suggests an intensive admixture and a high risk of full replacement of the Borgou population by AFZ genotypes. We observed similar AFT ancestral proportions in the historical Borgou (Bo_hist) and Adm_cur. The AFT ancestral proportion in the later population, comprising the Pabli samples, indicates the existence of a residue of the Beninese indigenous crossbreeds. According to Pecaud (1912) (as reported by Atchy, 1976), the Pabli breed results from crossbreeding between Borgou and Somba around the year 1905. In addition, the region of Kerou hosting the Pabli cattle in Western Benin is also less affected by transhumance. The association between spatial indicine introgression and transhumance in the Beninese cattle population was described by Scheper et al. (2020). Finally, the existence of crossbreed populations with divergent admixture levels offers the opportunity to evaluate the impact of crossbreeding in terms of divergence in extended haplotype homozygosity profiles.

### Selection Signatures

Previous studies (Lohmueller et al., 2010; Freedman et al., 2016) observed that admixture or further demographic events (population bottlenecks due to diseases) affect ancestral haplotypes and increase the occurrence of mosaics in the genome (Freedman et al., 2016; Aliloo et al., 2020). This may impede the distinction of genomic footprints left by neutral processes and natural selection. In consequence, we did not expect evidence for historical selections in the Beninese cattle population. Nevertheless, the selection is not dissociable from admixture in several African cattle breeds. Admixture is a historical practice in African cattle production and is considered as a quick means of animal upgrading (Flori et al., 2014). Researchers reported that the selection of the animal or the breed for crossbreeding is driven by farmer interests including desired productive (milk, meat, and reproductive) and adaptive features (left by natural selection) (Boutrais, 2007). In this context, the prevalence of specific genomic regions or functional traits within a population may reflect the production goals of the farmers. In addition, specific features in the genome of African cattle populations (including several populations with various crossbreeding histories) are commonly assessed with selection signature analyses (Gautier et al., 2009; Taye et al., 2018; Aliloo et al., 2020; Kim et al., 2020).

By contrasting the current and historical cattle populations in the Beninese taurine and crossbreeds, we focus on genomic footprints resulting from recent environmental pressures or herd management. Indeed, environmental and socioeconomic factors have induced diverse changes in cattle management practices in Benin. These include the adoption of cattle mobility in taurine or agropastoralist herds (former sedentary), the increase of herd mobility frequencies and amplitudes, the migration and settlement of several agropastoralists from Sahelian countries or Northern Benin into Southern Benin, and the extension of animal entrustment practices (from agropastoralists to traditional pastoralists) (Houessou et al., 2019a; Houessou et al., 2020; Vanvanhossou et al., 2021). Despite the limited period between our and the historical samples, we identified a few candidate regions, providing new insights into the evolutionary process in the indigenous breeds. In comparison to the taurine, we observed that the crossbreeds, especially BO_cur, displayed most of the candidate regions. These results are in line with increased Zebu introgression in BO_cur, leading to a higher admixture proportion than Adm_cur and more genetic divergence from the historical Borgou (BO_hist). Regarding the Somba (SO_cur), the identification of five candidate regions mainly associated with immunity features suggests the importance of disease pressures in its belt, as extensively discussed below.

Our approach to detect a strong signal of homozygosity considering temporal subpopulations within the same breed is similar to the one applied by Naderi et al. (2020), who contrasted the current German Holstein to one of its recent ancestors, the local dual-purpose German black pied cattle (DSN). In addition, we aimed to reduce the bias due to the uncertainty of the ancestral base population and applied the XP-EHH method to detect complete sweeps in contrast to the Integrated Haplotype Score (IHS) approach (Qanbari and Simianer, 2014). Indeed, the IHS method usually applied to investigate within-breed selection signatures relies on ancestral allele frequency which is inconsistently defined in different studies. For instance, Utsunomiya et al. (2013) derived ancestral alleles from common founders of Bovidae species, namely, *Bos gaurus*, *Bos grunniens*, and *Bubalus bubalis*. Kim et al. (2020) considered fixed alleles in African Buffalo as ancestral alleles to determine selection signatures in the African population, while Gautier et al. (2009) estimated ancestral alleles for West African cattle breeds based on alleles frequencies in indicines and African and European taurine samples. Finally, we expect to reduce false-positive results due to multiple tests, by considering adjusted *p-*values to define significant SNP and by defining only regions with at least three significant SNP as candidate regions. The respective candidate regions identified for each cattle population are discussed in the ongoing sections.

#### Lagune

We were not able to detect any candidate regions for DAH_cur. The Dahomey cattle may have acquired very few complete selective sweeps that are not detectable by the XP-EHH. This result reflects the breeding strategy with only focus on leisure and is in line with its high genomic inbreeding coefficient (http://www.dahomey-zwergrind.com). Further investigations including a complete sequencing of the Dahomey cattle and other methods of selection signature analyses may help untangle the genetic divergence between the Dahomey and the Lagune.

The admixed LA_out population was not considered for selection signature analysis because of the divergence in introgression levels as shown in the population structure analyses and admixture test. The LA_out group is the product of diverse admixed animals sampled as Lagune and therefore cannot represent any specific cattle population in Benin. On the contrary, LA_cur, as a relatively pure Lagune population, displayed three candidate regions. The region 15.5–16 Mb on BTA21 including the most significant SNP in LA_cur is identical to selection signatures reported in Holstein and North African cattle (Taye et al., 2017b; Ben-Jemaa et al., 2020). The *SV2B* gene in this region is associated with feed intake in cattle (Seabury et al., 2017). In contrast to the other populations investigated in this study, the candidate regions under selection in LA_cur encompassed relatively few genes functionally described in the literature. Nevertheless, the *WDR87* gene, hosting the most significant SNP, is related to carcass traits in cattle (Lim et al., 2013). In addition, the majority of the genes are involved in cellular and regulation of RNA biosynthetic processes, which are responsible for feed efficiency and body weight (Olivieri et al., 2016). Similarly, QTL associated with body weight, milk and reproductive traits are predominant in the candidate regions.

#### Somba

With regard to the five candidate regions under selection in SO_cur, two were detected on BTA23. This chromosome is known to have two subregions (classes IIa and IIb) of the bovine leukocyte antigen (BoLA), also called bovine major histocompatibility complex (MHC), which is determinant in the development of acquired immune responses to diverse parasitic and viral diseases (Ellis and Ballingall, 1999; Takeshima and Aida, 2006). The selective region 7–7.5 Mb (BTA23) spanning several genes (e.g., *DSB*, *BOLA-DYA*, *BOLA-DMB*, and *BOLA-DOA*) overlaps with the subregion of BoLA class IIb (Takeshima and Aida, 2006). These BoLA class IIb genes are specific to ruminants but are less characterized, in contrast to genes in the BoLA class IIa (e.g., *BoLA-DRB3* and *BoLA-DQA3*) (Takeshima and Aida, 2006). In addition, Ballingall and McKeever (2005) associated the rare polymorphism of BoLA class IIb genes to evolutionary processes under functional constraints. Selection signatures in this genomic region were only found in Angus and Brangus cattle (Goszczynski et al., 2018; Maiorano et al., 2018), whereas selection for adaptive immunity in African cattle breeds is usually identified within the BoLA class IIa region (Ballingall et al., 1997; Kim et al., 2017; Tijjani et al., 2019). Therefore, recent selection signatures in the specific BoLA class IIb genes may indicate a possible adaptation of the Somba cattle to endemic diseases, especially to anthrax. Indeed, the Somba cattle are affected by several recent episodes of anthrax outbreaks especially in the years 2007, 2009, 2012, and 2013 (Dossa and Vanvanhossou, 2016). Further studies are required to investigate the association between BoLA subregion IIb and resistance to disease in Somba and other African cattle breeds. In addition, other candidate genes include *PSMB9* and *HSD17B8* which are involved in meat and growth traits (Lee et al., 2012; Ma et al., 2015), *FSTL4* associated with milk production (Sanchez et al., 2019) and *ZFAND3* responsible for reproduction (Mohammadi et al., 2020). The predominance of candidate genes associated with immune response is confirmed by enriched terms such as antigen processing and presentation, and adaptive immune response. However, other biological processes include the regulation of cellular metabolic processes and intracellular protein transport mechanisms. The identified bovine QTL suggests selection on body weight, carcass, reproduction, and milk traits in the Somba cattle.

#### Crossbreeds

The regions of selection (54.5–55.5 Mb on BTA5, 100–100.5 Mb, and 101–101.5 Mb on BTA3) overlapping in the two hybrid populations are in line with their common indicine background. The significant SNP mapped in the region from 100 to 101.5 MB on BTA3 were positioned in various genes including the *RNF220* gene. This gene has been previously identified under selection in West African cattle (Gautier et al., 2009). In addition, the region includes genomic footprints of signatures in South African and East African Shorthorn hybrids with indicine ancestry deficiency in the later breed (Bahbahani et al., 2015; Zwane et al., 2019). These findings suggest that the region may represent an ancient and stable footprint of selection in indigenous African hybrids. The *RNF220* gene is involved in calving performance and milk yield (Abdel-Shafy et al., 2020; Purfield et al., 2020). In addition, the HECTD3 in this region is associated with cell cycle regulation and fat deposition, while PLK3 is related to gain and feed intake in cattle (Yu et al., 2009; Bahbahani et al., 2015; Zarek et al., 2017). We also detected in this region the PTCH2 and *SLC6A9* which are involved in reproduction and Porphyria disease, respectively (Nezamzadeh et al., 2005; Basavaraja et al., 2021). The region 55–55.5 Mb on BTA5 previously displayed evidence of selective sweeps in the EUT Charolais as well as in a tropical crossbreed between Charolais and Zebu, namely, the Canchim (Xu et al., 2015; Urbinati et al., 2016; Naval-Sánchez et al., 2020). Moreover, the *LRIG3* gene in this region is associated with body length in cattle and litter size in pigs (Xu et al., 2015; Metodiev et al., 2018).

Specific selection signatures detected in BO_cur include the selective sweep 46.5–47.5 Mb on BTA16. This region is of great interest. It presents several significant SNP and overlaps with genomic footprints detected in East African Zebu cattle as well as in a subpopulation of the German dual-purpose black and white cattle (Taye et al., 2018; Naderi et al., 2020). The region contains *DNAJC11*, a heat shock protein gene, involved in response to heat stress (Li et al., 2015). In addition, the candidate regions of selection (BTA 5:111.5–111.5 Mb and BTA 2:70–71 Mb) are known candidate regions under selection in different African, European and Asian cattle and sheep breeds (Hudson et al., 2014; Bomba et al., 2015; Wang et al., 2015; Bertolini et al., 2020). The region 111.5–111.5 Mb on BTA5 contributed to positive selection for natural virus resistance and to extensive admixture in West Sahelian African human populations (Cagliani et al., 2011; Pérez-Rivas et al., 2014; Triska et al., 2015). In addition, the segment BTA 2:70–71 Mb covered various candidate genes such as *EN1*, involved in growth traits in cattle (Buroker, 2014). Further regions under selection in BO_cur host candidate genes, significantly associated with different traits, including *ADAM32*, *ADAM9*, *HTRA4*, and *KLHL21* with residual feed intake and immune responses, *PLEKHA2* and *TNFRSF25* with growth and carcass performances, *HTRA4* with milk, and *HOOK1* with heat stress (Fan et al., 2015; Tizioto et al., 2015; Seong et al., 2016; Blanco et al., 2017; Hardie et al., 2017; Hay and Roberts, 2018; Sengar et al., 2018; Skibiel et al., 2018; Oliveira et al., 2019; Braz et al., 2020; Brunes et al., 2021; Soares et al., 2021).

Regarding the selection signatures in Adm_cur, the region 20–21 Mb on BTA4 in Adm_cur is consistent with the region reported by Naderi et al. (2020), who identified selection signatures in close distance to the *TMEM106B* gene in the German black pied cattle. In addition, the *ARL4A* gene was reported in the context of selective sweeps in Australian Holstein (Larkin et al., 2012) and with regard to copy number variations in African Nguni cattle and Polish Holstein (Wang et al., 2015; Mielczarek et al., 2017). The latter gene is associated with milk production in dairy (Raschia et al., 2018; Khan et al., 2020). The *SCIN* gene, also mapped in the region, is involved in residual feed intake in cattle (Salleh et al., 2017). The remaining regions under selection in Adm_cur spanned other candidate genes including *ANKRD52*, *RNF41*, and *MYL6* associated with height and carcass traits (Cai et al., 2019; Moravčíková et al., 2019; Feitosa et al., 2021), and *COQ10A* and *RNF41* related to milk trait and calf mortality (Lázaro et al., 2021; Marín-Garzón et al., 2021). In addition, the *SARNP* gene is related to animal reproduction (Labrecque et al., 2014), while the *DNAJC14* is involved in heat stress (Bahbahani et al., 2015; Rehman et al., 2020).

Overall, the common and specific candidate regions identified in the hybrid populations confirm selection signatures in African and European crossbreeds (Supplementary Table S6). They cover several candidate genes related to economic and functional traits. The enriched biological processes including inner mitochondrial membrane organization and ATP-dependent chromatin remodeling are related to carcass traits, milk production, and reproduction in cattle (Lu et al., 2016; Zhang and Xie, 2019; Shi et al., 2021). We also observed that the hybrids present more candidate regions related to heat response than the taurine populations, which is in line with the admixture with Zebu cattle known to tolerate high environmental heat loads (Taye et al., 2017a; Kim et al., 2020). Moreover, few genes in candidate regions of selection are involved in immune response and feed efficiency. The evidence of selection for adaptive traits in the hybrid populations (including BO_cur that is highly admixed) may be related to the fact that the AFZ introduced in the production environments of the West African taurine for decades have also developed various adaptive features (Atchy, 1976; Houessou et al., 2019b). Consequently, their crossbreeding with indigenous taurine cattle reduces the risk of diluting adaptive traits in local breeds while offering the opportunity to increase animal performances. Our findings are in line with those reported in other African cattle breeds (Aliloo et al., 2020; Kim et al., 2020) and suggest the ability to develop robust and productive breeds via crossbreeding. Nevertheless, the improvement of cattle breeding in West Africa requires the establishment of sustainable crossbreeding programs and the enhancement of genomic selection including genotype by environment interactions in the indigenous breeds. These will be achieved through the determination of suitable breeds and optimal proportions of admixture, considering social-ecological constraints (Wollny, 2003; Wurzinger et al., 2014).

## Conclusion

In this study, we confirm that the Dahomey cattle currently bred in Europe are a subpopulation of the Beninese indigenous Lagune breed. The high genomic inbreeding in the Dahomey population is due to its current breeding system. The introduction of new animals from zoological parks can increase the diversity of the Dahomey population. Moreover, the Beninese taurine indigenous Lagune and Somba cattle still conserve a high proportion of AFT ancestry, in comparison to the historical population. The Borgou displays a risk of full genetic replacement by African Zebu. However, we observed the existence of a hybrid population relatively less affected by ongoing indicine introgression and comparable to the historical Borgou.

We found no evidence of the negative impact of admixture on the adaptive features in the cattle populations including the crossbreeds, as they all present several genomic footprints involved in immune response, feed efficiency, and heat stress. Moreover, specific candidate regions in the Somba cattle demonstrate selection pressures related to endemic diseases in the habitat areas of the breed. Overall, identified recent selection in Beninese indigenous cattle towards productive traits such as reproduction, milk, and carcass traits favor the improvement of the economic merits of the breeds.

## Data Availability

All the data supporting the results of this article are presented within the article or in the additional files. The raw genotypic data used in this study are openly accessible at http://dx.doi.org/10.22029/jlupub-73.
